# Chickpea (*Cicer arietinum* L.) as a Source of Essential Fatty Acids – A Biofortification Approach

**DOI:** 10.3389/fpls.2021.734980

**Published:** 2021-10-12

**Authors:** Amod Madurapperumage, Leung Tang, Pushparajah Thavarajah, William Bridges, Emerson Shipe, George Vandemark, Dil Thavarajah

**Affiliations:** ^1^Plant and Environmental Sciences, Clemson University, Clemson, SC, United States; ^2^Agilent Technologies, Glasgow, United Kingdom; ^3^Grain Legume Genetics and Physiology Research Unit, Washington State University, Pullman, WA, United States

**Keywords:** chickpea (*Cicer arietinum* L.), essential fatty acids, biofortification, nutritional breeding, fourier transform infrared spectroscopy

## Abstract

Chickpea is a highly nutritious pulse crop with low digestible carbohydrates (40–60%), protein (15–22%), essential fats (4–8%), and a range of minerals and vitamins. The fatty acid composition of the seed adds value because fats govern the texture, shelf-life, flavor, aroma, and nutritional composition of chickpea-based food products. Therefore, the biofortification of essential fatty acids has become a nutritional breeding target for chickpea crop improvement programs worldwide. This paper examines global chickpea production, focusing on plant lipids, their functions, and their benefits to human health. In addition, this paper also reviews the chemical analysis of essential fatty acids and possible breeding targets to enrich essential fatty acids in chickpea (*Cicer arietinum*) biofortification. Biofortification of chickpea for essential fatty acids within safe levels will improve human health and support food processing to retain the quality and flavor of chickpea-based food products. Essential fatty acid biofortification is possible by phenotyping diverse chickpea germplasm over suitable locations and years and identifying the candidate genes responsible for quantitative trait loci mapping using genome-wide association mapping.

## Introduction

Chickpea (*Cicer arietinum*) is a self-pollinating diploid (2n=2x=16) pulse crop with a 738Mbp genome (Varshney et al., 2013). Chickpea primarily extended from *Cicer reticulatum* Ladizinsky approximately 11,000years ago (Zohari and Hopf, 2000; Kerem et al., 2007), a variable wild species that originated in several regions of southeastern Turkey (37.3–39.3°N, 38.2–43.6°E; Kerem et al., 2007). Chickpea presently has 44 species, of which 35 are perennial, and nine are annual. Chickpea has two market classes—*kabuli* and *desi—*based on seed morphology (Knights and Hobson, 2016). Kabuli has become popular in Western markets as hummus and canned and raw seeds for salads and soups, whereas desi seeds are split and consumed in Southeast Asia as “channa dal.”

Chickpea consumption is popular in many regions around the globe, mainly due to its high nutritional quality. The chickpea seed matrix is comprised of carbohydrates (50–58%), protein (15–22%), moisture (7–8%), fat (3.8–10.20%), and micronutrients (<1%; Jukanti et al., 2012; USDA, 2021). Chickpea carbohydrates include a range of prebiotic carbohydrates, including sugar alcohols, fructooligosaccharides, raffinose family oligosaccharides, inulin, and resistant starch (Peterbauer and Richter, 2001; Johnson et al., 2020), which modulate the gut microbiome and improve human gut health (Roberfroid et al., 2009). The mean protein content in chickpea is nearly 18% [(kabuli: 18.4% (16.2–22.4%); desi: 18.2% (15.6–21.4%)], which is higher than lentil and field pea (Upadhyaya et al., 2016). Chickpea is rich in lysine and arginine and low in sulfur (S)-containing amino acids such as cysteine and methionine (Jukanti et al., 2012). Moreover, chickpea is a rich source of minerals, including iron (Fe), zinc (Zn), and selenium (Se).

The United Nations established Sustainable Development Goals to end global hunger and malnutrition by 2030 (United Nations, 2021a,b). Biofortification or conventional breeding with modern biotechnology to enhanced micronutrient concentrations in staple food crops has been vital to combat global hunger and malnutrition. To date, many staple food crops have been biofortified with micronutrients, and cultivars were released to these vulnerable populations globally (Harvest Plus, 2021). Chickpea is a target candidate pulse crop for mineral and vitamin biofortification (Thavarajah and Thavarajah, 2012; Vandemark et al., 2018, 2020; Kumar and Pandey, 2020). During the last decade, several global research foundations have attempted to develop Fe-, Zn-, and Se-enriched chickpea cultivars to combat micronutrient malnutrition or “hidden hunger” (Thavarajah and Thavarajah, 2012; Vandemark et al., 2018). Biofortified chickpea provides 5.2–6.0mg of Fe, 2.5–5.3mg of Zn, and 15.3–56.3mg of Se in a 100-g serving, representing a significant portion of the recommended daily allowance (RDA) of these essential elements (Thavarajah and Thavarajah, 2012; Ray et al., 2014; Vandemark et al., 2018). A 100-g serving also provides 125–159mg of magnesium (Mg), 93–197mg of calcium (Ca), 0.7–1.1mg of copper (Cu), 732–1,126mg of potassium (K), and 263–370mg of phosphorus (P; Thavarajah and Thavarajah, 2012). Chickpea is also a significant source of carotenoids; beta-carotenoid is the most abundant, followed by canthaxanthin and xanthophyll (Thavarajah and Thavarajah, 2012). Vitamins such as folic acid, tocopherols, and vitamin B complex (B_2_, B_5_, and B_6_) are also found in chickpea (Jukanti et al., 2012). Overall, chickpea is a rich source of prebiotic carbohydrates, protein, and several micronutrients, and these components have already been incorporated into global chickpea biofortification programs (Thavarajah and Thavarajah, 2012; Vandemark et al., 2020). However, fat composition is the least-studied nutritional trait of chickpea, and genetic advancement studies are required to advance fatty acid biofortification.

Fats, which provide the storage energy required for seed germination (Nelson and Cox, 2008), occupy a minor proportion of the chickpea seed matrix compared to other nutrients. Chickpea is not an oilseed crop but has a higher fat content than other pulse crops (Jukanti et al., 2012). Sterols, tocopherols (phytosterols), and lipids are components of fat found in chickpea (Jukanti et al., 2012). The fatty acids in chickpeas—polyunsaturated fatty acids (PUFAs), monounsaturated fatty acids (MUFAs), and saturated fatty acids (SFAs)—mainly originate from the lipids. These are essential fatty acids (EFAs; ω-6 and ω-3 PUFAs), vital for humans in the biosynthesis of hormones and maintaining cellular integrity (Di Pasquale, 2009). Consequently, chickpea consumption can benefit human health by providing important fatty acids. This review focuses on global chickpea production, biofortification, the function of fats and benefits to human health, chemical analysis of EFAs, and possible breeding targets to optimize ω-6 and ω-3 fatty acids chickpea.

## Chickpea Production

Chickpea ranks third in the global production of pulses at ~11.6 million tons *per annum*, 80% of which is desi and the remaining 20% is kabuli (Merga and Haji, 2019). Chickpea is grown in nearly 57 countries worldwide in varying climatic and growing conditions (Merga and Haji, 2019). India was the leading global chickpea producer in 2019, followed by Turkey, Russia, Myanmar, Pakistan, and Ethiopia (FAOSTAT, 2020; Table 1). In great part due to India’s large-scale production, Asia dominated global chickpea production in 2019 compared to the Americas (83.4 vs. 6.1%, respectively; FAOSTAT, 2020). In the last two decades, the harvested area has correlated with chickpea production, and both generally show an increase over time (except for lower production in 2015 and 2019; FAOSTAT, 2020). Notably, India has lower yields than smaller producers such as Ethiopia and Mexico (FAOSTAT, 2020), resulting in its position as the world’s largest chickpea importer despite its large-scale production (Merga and Haji, 2019). During the last 2years, India’s imports increased from 0.19 MT in 2018 to 0.37 MT in 2019, possibly due to the lower yields in 2019 (9.93 MT) than in 2018 (11.3 MT).

**Table 1:** Global chickpea production and mean grain yields in 2019 (FAOSTAT, 2020).

Country	Production (MT)	Yield (kg/ha)
India	9.93	1,041
Turkey	0.63	1,217
Russia	0.51	918
Myanmar	0.49	1,316
Pakistan	0.45	474
Ethiopia	0.45	2084
United States of America	0.28	1730
Australia	0.28	1,069
Canada	0.25	1,614
Mexico	0.20	2,117

## Biofortification

Malnutrition is a persisting global calamity that is prevalent mainly in Africa and South Asia. It exists in three aspects: undernutrition (stunting, wasting, and underweight), obesity, and malnutrition associated with micronutrient deficiency (hidden hunger). The World Health Organization (WHO) estimates over 2 billion people suffer from hidden hunger (Ritchie and Roser, 2017). At the same time, 150.8 million, 50.5 million, and 38.3 million children aged below 5years are stunted, wasted, and overweight, respectively (Ritchie and Roser, 2017; Global Nutrition Report, 2018). South Asian women and school children are highly vulnerable to malnutrition. One-third of women of reproductive age are anemic and show higher susceptibility to obesity than men (Global Nutrition Report, 2018). Plant breeding and agronomical practices introduced in the 1960s during the green revolution primarily combatted global hunger, especially through large-scale cereal production, providing the necessary calories or proteins to these vulnerable populations (Thavarajah et al., 2014; Roorkiwal et al., 2021). However, the consumption of cereals contributed to hidden hunger or micronutrient malnutrition in most developing nations (Roorkiwal et al., 2021). Micronutrients mediate human physical and mental development and further serve as cofactors of enzymes that catalyze biochemical reactions in the body, modulating human physiology and growth (White and Broadley, 2005; Malik and Maqbool, 2020).

Several global approaches have been implemented to increase the bioavailability of nutrients in staple food crops (Welch and Graham, 2004; White and Broadley, 2005; Thavarajah and Thavarajah, 2012). However, technological, socio-economical, financial, and demographical constraints are challenges with nutrient fortification programs. Biofortification is breeding crops to optimize micronutrient concentration and bioavailability, enriching their nutritional value to combat hidden hunger (Garcia-Casal et al., 2017; Roorkiwal et al., 2021). Biofortification has three strategies: agronomic (fertilizing the soil or foliar application), conventional breeding approaches, and molecular technologies (Garcia-Casal et al., 2017). Biofortification is a convenient approach to combat ‘hidden hunger,’ primarily due to low financial investment, tendency to penetrate demographic barriers benefitting rural populations, and provision of germplasm to farmers at zero marginal expenditure during early investment (Bouis and Saltzman, 2017). In 2015, crops were biofortified for vitamin A (fleshy-orange sweet potato, cassava, and orange corn), Fe (beans and millet), and Zn (rice and wheat) by officially releasing the germplasm of biofortified varieties in 30 countries, further expanding trials and official breeding in more than 50 countries (Birol et al., 2015). However, biofortification attempts have been less frequent in pulses than in cereals (rice, wheat, and corn) during the present decade (Kumar and Pandey, 2020).

Current biofortification efforts in chickpea are focused on enriching micronutrients and reducing antinutrient factors (Sreeramaiah et al., 2007; Jukanti et al., 2012; Thavarajah and Thavarajah, 2012; Vandemark et al., 2020). Agronomic approaches such as fertilizer application (including foliar spraying) and genetic engineering (GE) have been attempted on chickpea to enrich minerals, such as Fe, Zn, and Se (Table 2, Poblaciones et al., 2014; Khalid et al., 2015; Pal et al., 2019, 2021). Soil and foliar application of Zn and urea can increase chickpea’s Zn and Fe content (Pal et al., 2019). A combined application of Fe, Zn, and urea (in a tank mix) can increase Fe and Zn concentrations in chickpea seeds and positively influence grain yields and protein levels (Pal et al., 2021). Two separate studies indicate inoculating Zn-solubilizing bacteria (*B. altitudinis*) and rhizobacteria at chickpea planting increase seed Zn and Fe concentration in low Zn soils (Khalid et al., 2015; Kushwaha et al., 2021). Transgenic approaches have also been used for Fe biofortification in chickpea. For example, overexpression of the nicotamine synthase 2 (CaNAS2) and ferritin (GmFER) genes in chickpea increased seed Fe concentration (Tan et al., 2018). However, the above transgenic approach has not demonstrated any significant outcome for conventional chickpea breeding programs. Although biofortification significantly focuses on micronutrients, the techniques followed can be applied to other macro nutritional traits (Garcia-Casal et al., 2017; Roorkiwal et al., 2021). Linoleic acid (LA; ω-6) is the most abundant (essential) fatty acid in chickpea, while α-linolenic acid (ALA; ω-3), the other essential fatty acid, is far less available in the seed (Jukanti et al., 2012). ALA is known for its human health benefits (Simopoulos, 2002, 2006, 2016). Thus, breeding chickpea to enrich the seed in ALA is important; however, the quantitative nature of these genetic traits makes chickpea breeding much complicated than for traits controlled by a single gene.

**Table 2:** Biofortification methods for chickpea.

Nutrient	Approach	Positive response	References
Selenium (Se)	Foliar application	Seed Se	Poblaciones et al., 2014
Iron (Fe)	Soil application of Plant growth-promoting rhizobacteria	Soil and seed Fe	Khalid et al., 2015
Zinc (Zn)	Foliar application with Zn fertilizer	Seed Zn	Shivay et al., 2015; Pal et al., 2019
Boron (B)	Seed coating	Nodulation, yield	Hussain et al., 2020
Fe and Zn	Conventional breeding/selection/backcrosses	Seed Fe and Zn	Singh et al., 2021

## Chickpea Lipids

**Figure 1:**
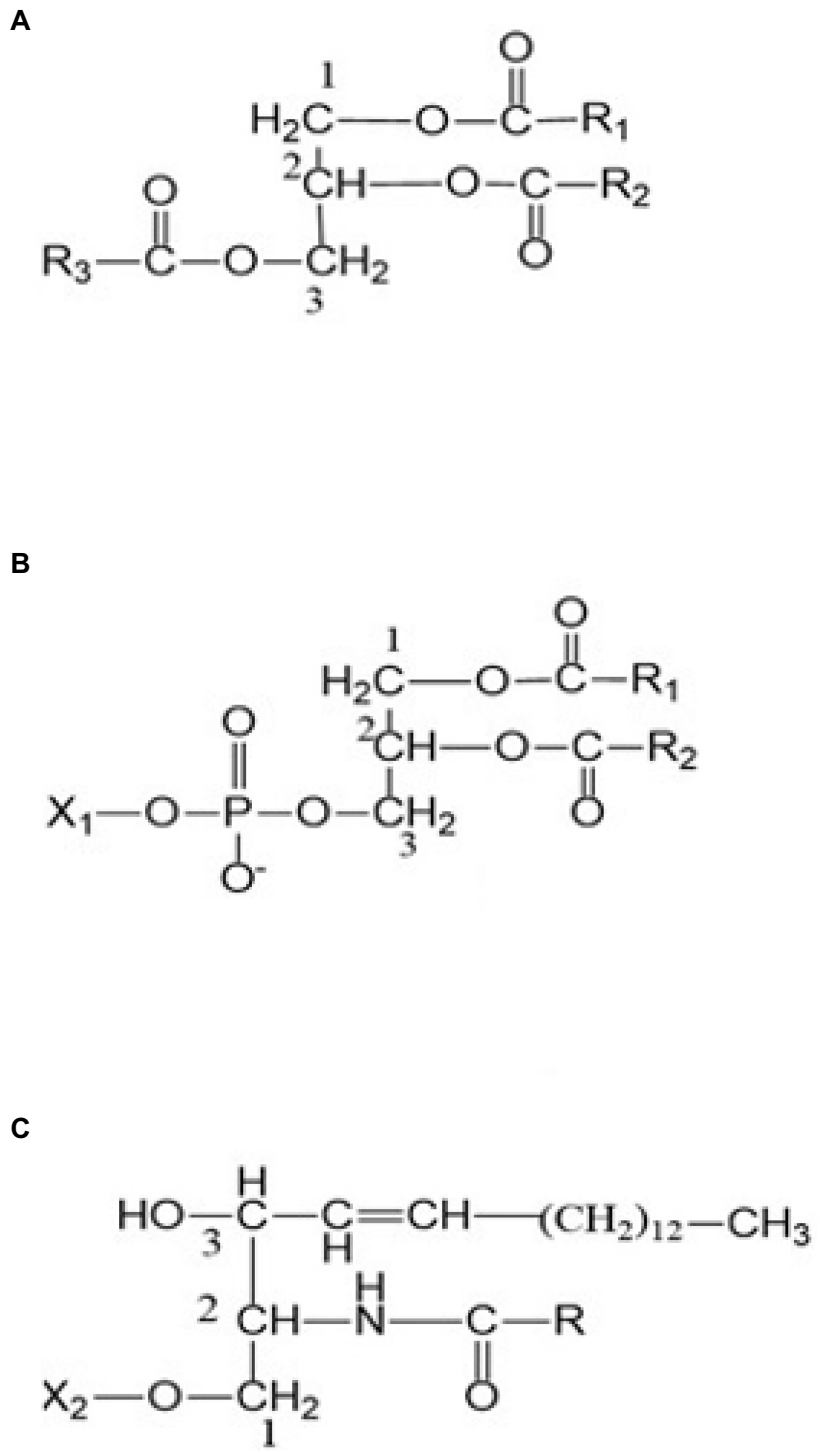
**(A)** A triacylglycerol (TAG) **(B)** a phospholipid, and **(C)** a sphingolipid. R, R_1_, and R_2_ are alkyl or alkenyl groups attached to ester carbonyls. X_1_: H, ethanolamine, choline, serine, glycerol, or phosphatidylcholine functional groups. X_2_: H, phosphocholine, glucose, or oligosaccharide functional groups.

**Figure 2:**
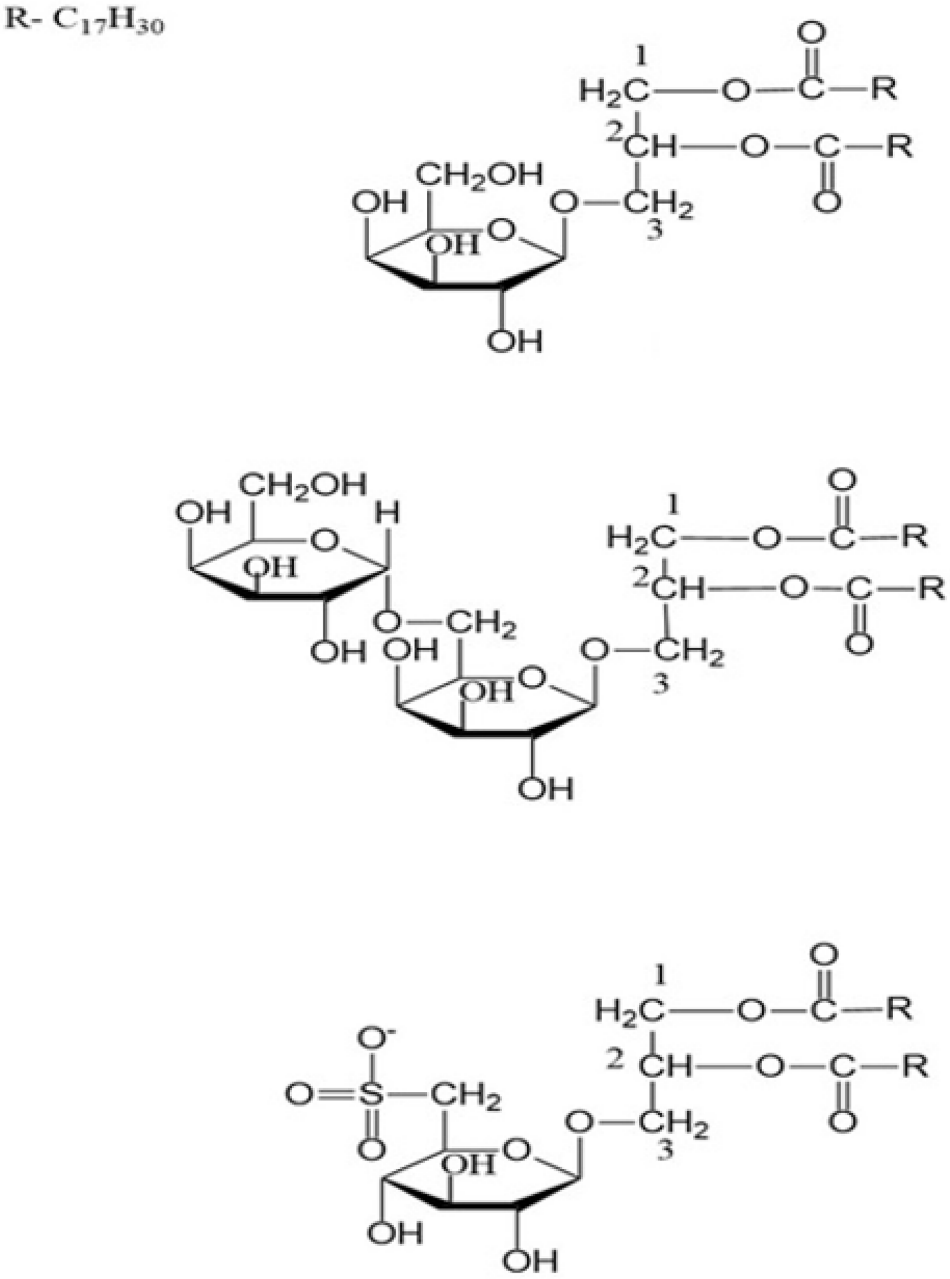
Some typical structures of galactolipids.

In chickpea, lipids persist as storage and membrane molecules. Storage lipids are triacylglycerols (TAGs), which are suspended as oil droplets (oily phase) on the cell cytosol serve as primary sinks of fatty acids (including EFAs; Nelson and Cox, 2008; Cagliari et al., 2011). TAGs are the most abundant neutral lipid in desi-type chickpea and typically serve as biosynthetic precursors and energy supplements during seed germination (Zia-Ul-Haq et al., 2007; Jukanti et al., 2012; Weselake et al., 2021). The general structure of TAGs includes a glycerol group esterified with three fatty acids, either similar or different (Figure 1A). Chickpea also has phospholipids (glycerophospholipids; Figure 1B), sphingolipids (Figure 1C), glycolipids (galactolipids and sulpholipids; Figure 2), and phytosterols as membrane lipids (Clemente et al., 1998; Zia-Ul-Haq et al., 2007; Michaelson et al., 2016). Both storage and membrane lipids contribute to the total chickpea fat composition. The majority of the fatty acids in chickpea are originated from the storage lipids (TAGs), which are the most abundant neutral lipids in seeds (Jukanti et al., 2012). Chickpea has a general fat content of 3.8–10.2%, which is higher than other pulse crops (e.g., lentils, red kidney beans, etc.; Jukanti et al., 2012); the fat content also varies with market class, with ranges from 3.4–8.8% and 2.9–7.4% for kabuli and desi, respectively (Yadav, 2007).

## Fatty Acids and EFAs

**Figure 3:**
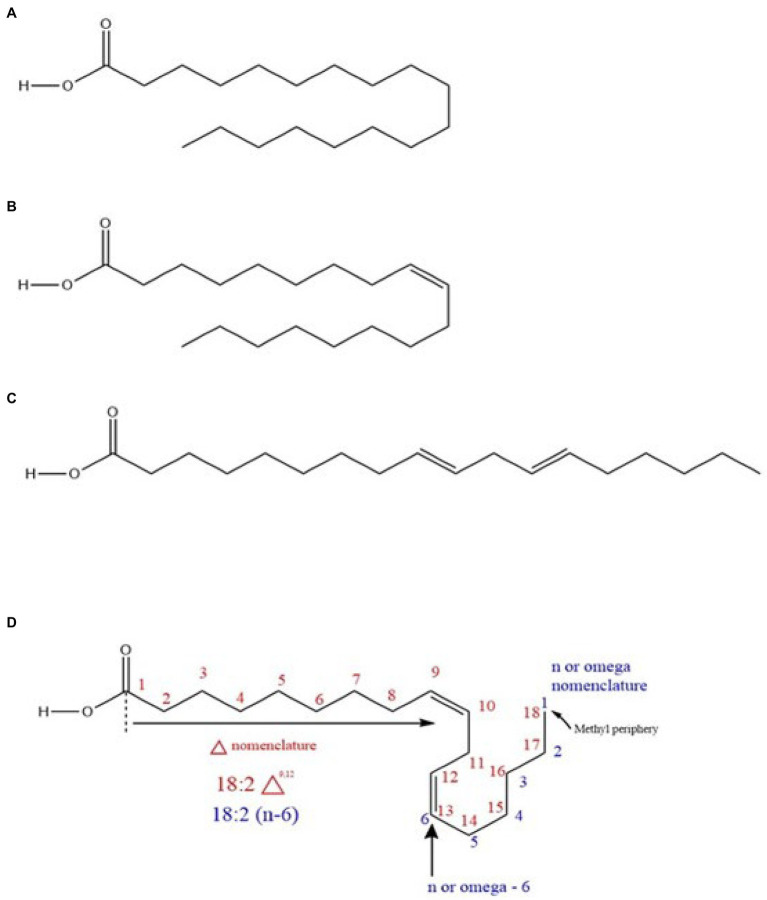
**(A)** A saturated fatty acid (stearic acid) **(B)** a monounsaturated fatty acid (MUFA; oleic acid) **(C)** a polyunsaturated fatty acid (PUFA; trans linoleic acid), and **(D)** nomenclature system of a PUFA (cis linoleic acid).

Typically, fatty acids are long-chain hydrocarbon molecules with an attached carboxylic acid group. In chickpea, fatty acids mainly originate from TAGs (Zia-Ul-Haq et al., 2007; Jukanti et al., 2012) as previously indicated and are classified as saturated (with double bonds) or unsaturated (no double bonds) based on the bonding nature (Fahy et al., 2005; Rustan, 2005; Figure 3). Unsaturated fatty acids are divided into PUFAs and MUFAs. LA (ω-6) and ALA (ω-3) are PUFAs (Innis, 1991), while oleic acid (OA; ω-9) is a MUFA. LA and ALA are EFAs because they are not synthesized in humans (animals) and must be supplemented from the diet, while OA is not (because animals produce it; Warude et al., 2006) but serves as a precursor for LA. The enzymes to convert OA to LA and then LA to ALA (12-desaturase and 15-desaturase, respectively) exist in plants (Warude et al., 2006; Lee et al., 2016, i.e., chickpea). Within total chickpea fats, 66% are PUFAs, 19% are MUFAs, and 15% are SFAs. Both market classes have considerable amounts of LA (kabuli: ~51.2%, desi: ~61.62%) and OA (kabuli: ~32.6%, desi: ~ 22.31%), which are generally higher than for other edible pulses such as lentils (LA: ~44.4%, OA: ~20.9%), beans (LA: ~46.7%, OA: ~28.1%), and peas (LA: ~45.6%, OA: ~23.2%; Wang and Daun, 2004). Chickpea also contains palmitic acid (kabuli: ~9.41%, desi: ~9.41%) and ALA (kabuli: ~2.69%, desi: ~3.15%; Wang and Daun, 2004; Jukanti et al., 2012).

## Fatty Acids and Human Health Benefits

A chickpea-based diet provides significant EFAs; the consumption of unsaturated vs. saturated fats can help maintain healthy cholesterol levels and reduce obesity and diabetic conditions (Kaur and Prasad, 2021). Furthermore, the presence of ALA in a chickpea-based diet reduces angiotensin-converting enzyme inhibition, which contributes to antihypertensive effects (Ogawa et al., 2009; Kaur and Prasad, 2021). Once EFAs are ingested, LA is metabolized to arachidonic acid (AA, an ω-6 EFA). In contrast, ALA is metabolized into eicosapentaenoic acid (EPA, an ω-3 EFA) and docosahexadecaenoic acid (DHA, an ω-3 EFA). AA and EPA undergo further biosynthesis to prostanoids and leukotrienes (de Caterina et al., 2007). These metabolites have several beneficial physiological effects on humans (Singh, 2005). Metabolites with an ω-6 origin enhance platelet aggregation, while those of ω-3 origin are anti-inflammatory (Singh, 2005). The ω-6/ω-3 fatty acid ratio is an important indicator of the impact of EFAs on human health (Simopoulos, 2002). This ratio is a disease-controlling parameter, where the optimum range is 1–4:1 or 1–5:1 (Simopoulos, 2002, 2006; Singh, 2005). This value ranges from 1–2:1 for optimum health benefits for combating obesity (Simopoulos, 2016). However, in Western countries, this value ranges from 15–16.7:1 due to the low levels of ω-3 fatty acids in diets and comparatively high proportions of LA consumption (Simopoulos, 2002). Yet, no studies regarding the true impact of chickpea on this disease controlling parameter (ω-6/ω-3 ratio) and human metabolism have been published.

Chickpea based diet has a positive effect on diabetes and obesity. Adiponectin is a hormone that prevents type two diabetes and atherosclerosis (Achari and Jain, 2017). A randomized cross-over clinical trial with diabetic patients (*n*=32) served with a chickpea diet (substituting two servings of red meat) increased levels of adiponectin in all patients (Mirmiran et al., 2019; Acevedo Martinez et al., 2021). Additionally, a study with diabetic rats has demonstrated reduced blood glucose and triglyceride levels upon feeding 400mg/kg of aqueous and methanol-based doses of chickpea diets (Yagi and Yagi, 2018). Another clinical study (n=30; men =17 and women=13) reported that body weights, systolic blood pressure, low-density lipoprotein (LDL), high-density lipoprotein (HDL), and total cholesterol reduced with a diet rich in chickpea and other legumes (Gupta et al., 2017). The above changes were significantly comparative to a diet restricted with legumes (Gupta et al., 2017). The effects of chickpea on obesity have been further studied using rats for 8months. Their study has included a fatty diet as control and control with 10% (w/w) chickpea. The results indicated a 35% increment in HDL whereas a 23% decrement in LDL with an overall 30% reduction in LDL/HDL ratio (Gupta et al., 2017). The efficacy of a chickpea-based diet on diabetes and obesity needs further investigation with extensive clinical studies for the long term. Few studies indicated that nutritional responses in pulses may have been due to its high levels of low digestible carbohydrates, proteins, micronutrients, and low in anti-nutrients such as phytic acid, amylase inhibitors and lectins (Thavarajah and Thavarajah, 2012; Gupta et al., 2017).

## The Impact of Food Processing on Fatty Acids

The fatty acid composition of chickpeas is sensitive to food processing. Cooking can increase the fat content in both kabuli and desi varieties (Wang et al., 2010), but pressure cooking can reduce the levels of the four main fatty acids in chickpea flour (Rajni et al., 2012; Table 3). Furthermore, food processing affects the quality and quantity of chickpea EFAs, as unsaturated fatty acids are directly exposed to oxygen and other reactants leading to auto-oxidation (Damodaran and Parkin, 2017). In particular, PUFAs are highly susceptible to auto-oxidation because they have more double bonds, any one of which could react with oxygen radicals (Damodaran and Parkin, 2017). Alkyl radicals with a PUFA origin are the major reactants that initiate PUFA depletion. High-temperature conditions in food processing could further increase these food quality-degrading reactions. Heat can significantly decompose the radicals formed (hydroperoxyl radicals) and multiply PUFA depletion (Damodaran and Parkin, 2017). The alterations depicted in Table 3 result from such chemical changes while cooking (Rajni et al., 2012; Damodaran and Parkin, 2017). The presence of certain minerals (especially Fe) and isoenzymes such as lipoxygenase in raw chickpea (Halliwell and Gutteridge, 1990; Sanz et al., 1992; Girotti, 1998; Damodaran and Parkin, 2017) may catalyze EFA depletion during storage. Lipoxygenase mainly contributes to depleting ALA and LA, initiating hydroperoxide formation (Damodaran and Parkin, 2017). As a result, storage conditions must inhibit lipoxygenase in the chickpea to preserve the food quality and enhance the shelf life. Another impact of auto-oxidation is forming volatile aldehydes and unsaturated by-products with rearranged double bonds (trans fats; Damodaran and Parkin, 2017). Trans fat formation from PUFAs could occur due to unsaturated double bond cleavage and rearranging during higher temperature food processing. Volatile compounds (aldehydes) formed due to storage and food processing deplete the quality and aroma, leading to rancidity (Damodaran and Parkin, 2017), while trans fats are detrimental to human health. However, no studies related to rancidity and trans fats originating from chickpea foods are available in the literature. Future studies are required to understand these fatty acid concentrations after processing, cooking, and storage.

**Table 3:** Fatty acid composition of raw and processed chickpea (Rajni et al., 2012).

Treatment	Fatty acid (%)			
	Palmitic acid	Oleic acid	Linoleic acid	Linolenic acid
Raw seed	9.7	27.9	57.3	1.6
Boiling	10.8	33.4	51.3	trace
Pressure cooking	9.6	27.7	56.3	1.6
Roasting	10.1	28.2	50.1	1.2

## Fatty Acid Analysis

Fatty acid extraction procedures and analytical instrumentation are essential for the accurate quantification of EFAs. Fatty acid profiles are measured using gas chromatography (GC) paired with a flame ionization detector (FID) or a mass spectrometer (MS; Laakso and Hiltunen, 2009; Chiu and Kuo, 2020). The FID is a universal detector, which creates signals for organic molecules (due to C-H bond cleavage), but fails in molecular identification (qualitative analysis; Skoog et al., 2018). Accordingly, MS is the most superior detection method for qualitative and quantitative analysis of fatty acids by GC. The advantage of a mass-based detector is the ability to run a selective ion monitoring (SIM) analysis for all analytes (Sleeman and Carter, 1997). The SIM mode enables quantification irrespective of two analytes having close retention times. FID detection requires tedious efforts in terms of temperature programming to obtain entirely resolved chromatograms with minimal errors. Therefore, MS with SIM is the most appropriate and convenient method for fatty acid quantification and identification (Sleeman and Carter, 1997; Dodds et al., 2005). However, a major drawback of GC–MS techniques is the analysis time, cost, and labor. A short analysis time with high throughput is ideal for collecting data to screen fatty acids in breeding populations before advancing to varietal development stages.

Fourier-transform infrared (FTIR) spectroscopy measures the infrared spectrum of absorption or emission of a solid, liquid, or gas (Sindhu et al., 2015) and is a suitable technique to reduce the analytical time, cost, and labor but preserve high throughput. FTIR data models validated with GC–MS methods are robust tools to quantify fatty acids for high-throughput plant breeding research (Gómez-Caravaca et al., 2013). Non-destructive sample preparation and the application of hand-held FTIR devices in the field will enhance future chickpea breeding to select for fatty acid-rich accessions without the need for an analytical laboratory.

The electromagnetic spectrum’s IR region is less energetic compared to the ultraviolet (UV)-visible region. Consequently, IR energy induces molecular vibrations rather than electronic excitations. The midsection of the IR (mid-IR, MIR) region has the most fundamental resonant frequencies that cause distinct molecular vibrations (Skoog et al., 2018). Consequently, FTIR utilizes MIR energy to generate signals based on molecular vibrations for qualitative and quantitative analysis. The working window of FTIR is 500–4,000cm^−1^, wherein signals due to functional group vibrations occur mainly between 1,500 and 4,000cm^−1^ (functional group region; Lohumi et al., 2015). For fatty acids (Figure 3), the C=O (carbonyl), C=C (unsaturated double bonds), and C-H bonds undergo distinct oscillations in the functional group region. Therefore, based on the signal intensities (C=C and C-H signal ratios), fatty acids can be characterized by the degree of saturation and chain length, followed by quantification (Meiklejohn et al., 1957; Rabelo et al., 2015). Carbonyl stretching (~1742–1750cm^−1^) is one of the most distinct signals and is strongly applicable to fatty acid quantification (Yang et al., 2005; Rabelo et al., 2015). FTIR is a powerful tool for fatty acid analysis. NIR (near-IR spectroscopy) is another common tool in plant breeding programs used in parallel with FTIR technology (Downey, 1999). NIR technology is also available with added Fourier-transformation technology (i.e., FTNIR; Skoog et al., 2018). The primary difference between FTNIR and FTIR techniques is the working window. The FTNIR range (4000–12,820cm^−1^) is beyond the MIR range employed in FTIR, and the molecular vibrations occur as overtones and combined bands in the NIR range (Yang et al., 2005; Lohumi et al., 2015; Rabelo et al., 2015). FTIR and FTNIR have both been used for total fat analysis in food and seed composition analysis, and each method has advantages and disadvantages. Generally, calibration models for total fat based on FTNIR are better than those based on FTIR (Yang et al., 2005; Oliveira et al., 2006); however, FTIR is more informative than FTNIR due to its well-resolved spectral signals and because it provides better qualitative insight (Lohumi et al., 2015). In addition to fat analysis, NIR spectroscopy has been used in routine seed composition analysis for moisture, protein, starch, kernel hardness, color, and seed viability (William and Norris, 2001; Kusumaningrum et al., 2018; Skoog et al., 2018). FTNIR spectroscopy fits well with quantitative measurements of compounds with functional groups containing C-H, N-H, and O-H bonds based on NIR vibrational overtones (Skoog et al., 2018). Furthermore, the qualitative identification of functional groups using NIR is not optimal due to low resolution (William and Norris, 2001). Overall, the FTIR technique is unique and accurate with good resolution as a high-throughput tool to measure individual nutritional trails with low concentrations.

## Breeding Approaches

Current chickpea breeding is mainly focused on grain yield, disease resistance, and nutritional quality traits, including protein, minerals, prebiotic carbohydrates, and environmental stresses (Wang et al., 2017; Vandemark et al., 2018, 2020). Seed yield can be positively or negatively correlated with chickpea agronomic traits. For example, Toker (2009) shows chickpea seed yield is positively correlated with biomass (*r*=0.975), harvest index (*r*=0.935), plant height (*r*=0.853), number of branches (*r*=0.797), and pods per plant (*r*=0.675) but negatively correlated with seed weight (*r*=−0.660) and ascochyta blight infection (*r*=−0.872). Wang et al. (2017) show positive correlations between seed protein concentration, plant height, and days of maturity and negative correlations between seed protein concentration, grain yield, and seed size. The concentrations of minerals, including K, P, and Zn, in chickpea seeds are influenced by genotype, location, and genotype×location interaction (Vandemark et al., 2018). Chickpea prebiotic carbohydrate concentrations vary across location, year, and genotype (Vandemark et al., 2020). Chickpea grain yield is negatively correlated with several prebiotic carbohydrates, including verbose (*r*=−0.80), stachyose (*r*=−0.77), sorbitol (*r*=−0.66), and mannitol (*r*=−0.65; G. Vandemark et al., 2020). Overall, grain yield is negatively correlated with most nutritional traits, including protein content, certain prebiotic carbohydrates, and minerals (Vandemark et al., 2018, 2020).

**Figure 4:**
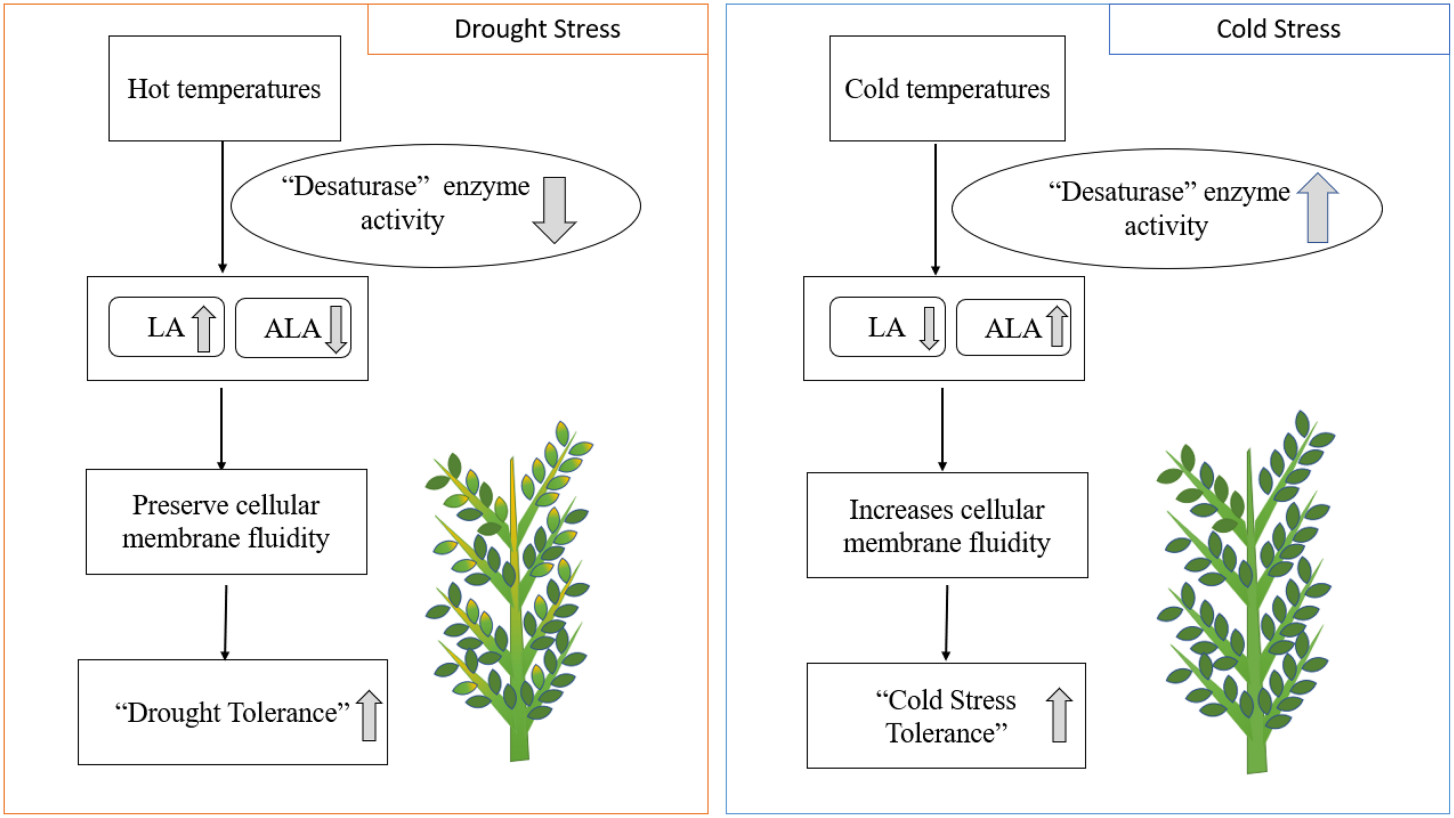
Drought and cold stresses impact enzymatic activity and EFA composition in plants. LA; linoleic acid. ALA; alpha-linolenic acid.

Heat, drought, and cold stresses are the common abiotic stresses affecting chickpea production worldwide (Jha et al., 2014). Plant lipids are linked to increased cold and heat tolerance in food crops. Fats alleviate environmental stresses by changing their PUFA composition in chloroplast lipids (Nelson and Cox, 2008). Drought stress generally increases LA and decreases ALA concentrations in response to desaturase enzymes (Yordanov et al., 2000). Lipids, including phospholipids and glycolipids, help chickpea plants withstand cold stress during the winter (Vigh et al., 1998). Desaturation of fatty acids is positively correlated with preventing cell lysis at colder temperatures (Bakht et al., 2006; Shah et al., 2013). The increase in double bonds in PUFA chains contributes to plant cell membrane fluidity, increasing cold tolerance due to freezing point depression (Vigh et al., 1998). Increased ALA and reduced LA levels in chickpea leaves during cold stress indicate fatty acid desaturation at low temperatures (Bakht et al., 2006). Higher double bond indices (DBI) in extracted leaf fats at negative LT_50_ (lethal temperatures) values indicate higher levels of unsaturated fats at lower temperatures (a significant negative correlation, *r*<0; Bakht et al., 2006; Figure 4). Genomic and gene-editing technology may enhance PUFA desaturation and accelerate breeding efforts to develop chickpea cultivars resistant to cold stress (Jaglo-Ottosen et al., 1998; Gilmour et al., 2000; Bakht et al., 2006). PUFA-induced mutations in chickpea have revealed higher PUFA (LA) content leads to improved cold stress tolerance (Shah et al., 2013). Mutant desi genotypes (CM72/02 and CM137-01) and mutant genotypes of desi×kabuli introgression can also tolerate sustained cold stress (Shah et al., 2013).

Integrating traditional breeding and biotechnology approaches would benefit the development of chickpea cultivars resilient to climate change. Planting time and growing conditions also affect fatty acid composition in chickpea seeds, with OA and LA concentrations higher in chickpea planted in the fall than in the spring (Gül et al., 2008). Interactions between genotype and planting date can significantly affect the concentration of α and β tocopherols and palmitic acid, OA, and LA concentrations; ALA concentration is positively correlated with LA concentration and negatively correlated with OA and tocopherol concentrations (Gül et al., 2008). Nine candidate genes related to fats have been identified in soybean using quantitative trait loci (QTL) mapping (Nian and Cheng, 2020). A single gene associated with a lipid synthesizing and storage enzyme named diacylglycerol O-acyltransferase has also been identified in chickpea (Verma et al., 2015). Detailed QTL mapping studies on candidate genes associated with essential fatty acids in chickpea have not been reported.

## Conclusion and Future Prospects

**Figure 5:**
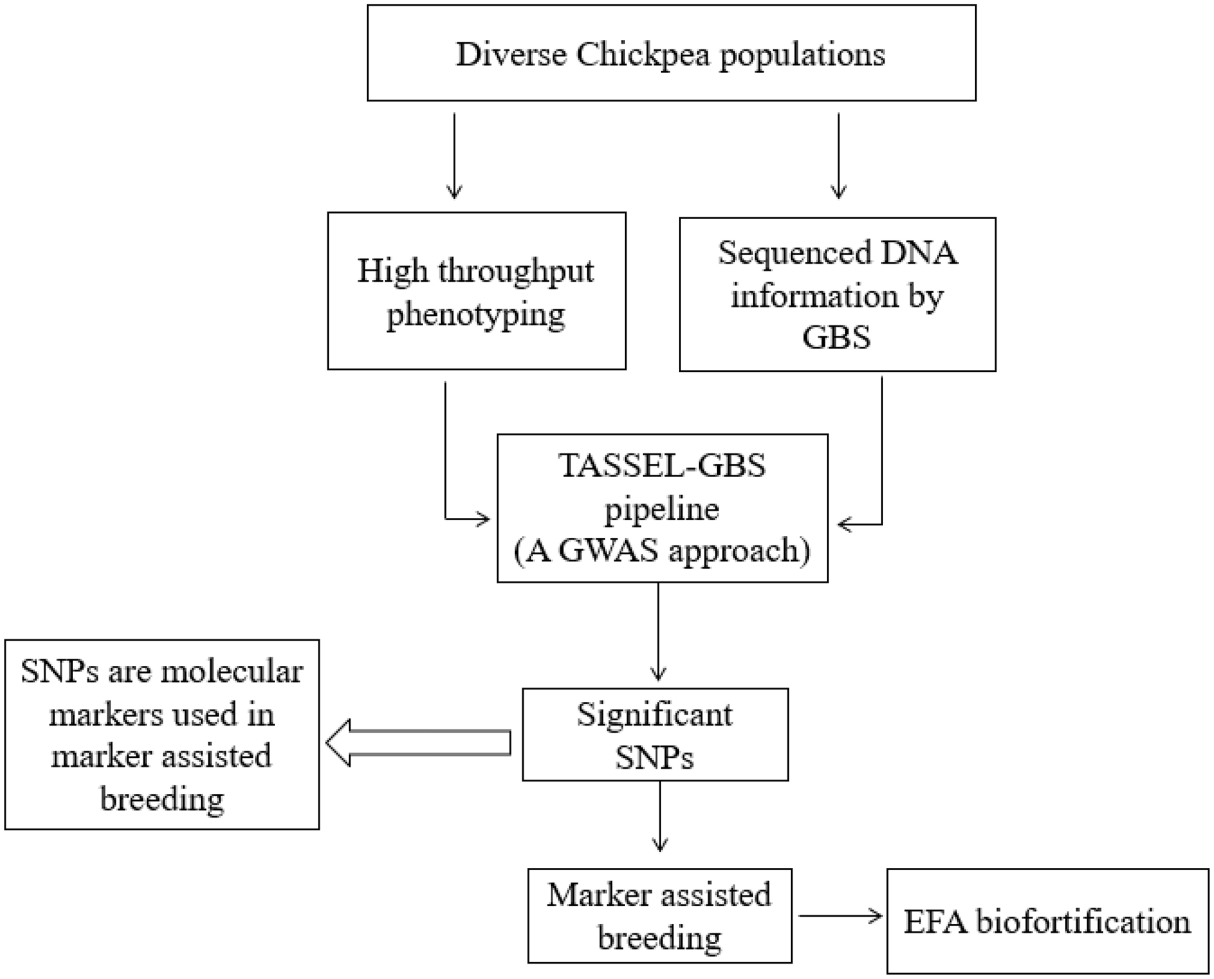
Genetic biofortification of EFA in chickpea.

Chickpea is a highly nutritious pulse crop rich in protein, prebiotic carbohydrates, fat, and a range of micronutrients. Chickpea is a rich source of EFAs, phytosterols, TAGs, and phospholipids. TAGs are the most dominant neutral lipid in chickpea. PUFAs, MUFAs, and SFAs are esterified within the lipids and bind to TAG’s glycerol end or a phospholipid. The most dominant PUFA in chickpea is LA, followed by OA (MUFA) and ALA. LA is an ω-6 EFA, whereas ALA is an ω-3 EFA. The consumption of diets with an ω-6/ω-3 ratio of 4 to 5 is recommended for better human health. The ratio of EFAs from a chickpea diet and related human health benefits have yet to be studied using large clinical trials. EFA traits have not been extensively studied in chickpea breeding. Optimizing EFA levels in chickpea should be feasible by applying the genetic and transgenic approaches followed in chickpea biofortification for micronutrients. FTIR and FTNIR techniques should be incorporated into breeding programs to screen breeding populations; FTIR within the functional group region will assist qualitative and quantitative fatty acid analysis. Future genome-wide association studies are needed to develop marker-assisted breeding approaches for improving chickpea nutritional traits. Genome mapping studies could support the identification of corresponding QTLs and candidate genes associated with fatty acid biosynthesis (Figure 5). In general, chickpea produces LA (2.87g/100g) and ALA (0.112g/100g), but the cultivar information is not available (USDA, 2021). So far, human clinical studies have been published to confirm the impact of the prevailing chickpea EFA composition on human health. Percent recommended daily allowance (%RDA) for LA (ω-6 fatty acid) is not published; however, the %RDA of ALA (ω-3 fatty acid) for adult men and women is 1.6 and 1.1g, respectively (Hjalmarsdottir, 2019). Future chickpea breeding strategies should address the safe, adequate increase of these essential fatty acids for human health. Future genomics and plant breeding advancements will also enhance chickpea’s EFA concentrations and other nutritional traits and improve human health.

## Author Contributions

AU is a doctoral graduate student working on this project with DT; they created the hypothesis, objectives, outline the draft, and wrote the manuscript. LT, PT, WB, ES, and GV edited and added discipline-specific feedback. All authors contributed to the article and approved the submitted version.

## Funding

This project was supported by the American people *via* the Feed the Future Innovation Lab for Crop Improvement through the United States Agency for International Development (USAID, award no 7200AA19LE00005/subaward no 89915-11295 awarded to DT); the Pulse Health Initiative (USDA-ARS awarded to DT and GV); and the USDA National Institute of Food and Agriculture, [Hatch] project [1022664] awarded to DT. Its contents are solely the responsibility of the authors and do not necessarily represent the official views of the USDA, USAID, or the United States Government.

## Conflict of Interest

The authors declare that the research was conducted in the absence of any commercial or financial relationships that could be construed as a potential conflict of interest.

## Publisher’s Note

All claims expressed in this article are solely those of the authors and do not necessarily represent those of their affiliated organizations, or those of the publisher, the editors and the reviewers. Any product that may be evaluated in this article, or claim that may be made by its manufacturer, is not guaranteed or endorsed by the publisher.
